# Enhancing Sensorimotor Activity by Controlling Virtual Objects with Gaze

**DOI:** 10.1371/journal.pone.0121562

**Published:** 2015-03-23

**Authors:** Cristián Modroño, Julio Plata-Bello, Fernando Zelaya, Sofía García, Iván Galván, Francisco Marcano, Gorka Navarrete, Óscar Casanova, Manuel Mas, José Luis González-Mora

**Affiliations:** 1 Department of Physiology, School of Medicine, University of La Laguna, Santa Cruz de Tenerife, Spain; 2 Department of Neurosurgery, University Hospital of the Canary Islands, Santa Cruz de Tenerife, Spain; 3 Centre for Neuroimaging Sciences, Institute of Psychiatry, King’s College London, London, United Kingdomm; 4 Laboratory of Cognitive and Social Neuroscience (LaNCyS), UDP-INECO Foundation Core on Neuroscience (UIFCoN), Diego Portales University, Santiago de Chile, Chile; University of Montreal, CANADA

## Abstract

This fMRI work studies brain activity of healthy volunteers who manipulated a virtual object in the context of a digital game by applying two different control methods: using their right hand or using their gaze. The results show extended activations in sensorimotor areas, not only when participants played in the traditional way (using their hand) but also when they used their gaze to control the virtual object. Furthermore, with the exception of the primary motor cortex, regional motor activity was similar regardless of what the effector was: the arm or the eye. These results have a potential application in the field of the neurorehabilitation as a new approach to generate activation of the sensorimotor system to support the recovery of the motor functions.

## Introduction

Physical therapy is a common treatment for motor deficits but it is not always possible because of limitations in the affected limbs. Thus, alternative approaches have been used to support the recovery of motor functions by generating an activation of the sensorimotor system without resorting to overt voluntary movements [1]. One approach is based on passive movements caused by an external agent (e.g. a physiotherapist). Such movements are thought to activate the sensorimotor system via proprioceptive afferences that input information to both motor and sensory regions [2,3]. Another approach is based on motor imagery, i.e. the mental rehearsal of motor acts in the absence of actual movement production. It is thought that motor imagery activates the motor system because it shares the same neural mechanisms that are involved in motor control of actual actions [4], providing additional benefits to conventional therapies [5]. There is a third approach, known as action observation therapy, which is only based on visual stimuli and uses the properties of the parietofrontal mirror neuron system. Such a system is not only activated when individuals perform a particular action but also when they observe others performing the same action [6–8]. Thus, the visual presentation of actions may facilitate the reorganization of the motor areas affected by stroke, and has shown good therapeutic results [9–12].

The three above mentioned ways of increasing sensorimotor activity involve a limb (real, imagined or observed) that performs movements in some way. However, activity in sensorimotor regions can be also produced without directly resorting to the movements of a limb: it is known that just with eye movements, activations are obtained both in the frontal and parietal cortices [13,14]. This cortical activity is involved in the control of ocular movements, while the execution of such movements relies, in a lower level, on the brainstem motor centers [15].

It is notable that the above mentioned cortical mechanisms are not only related to the control of the gaze but can be also related to the control of limb movements, as has been reported in many imaging studies outside the field of rehabilitation. For example, when reaching and saccade movements towards static targets have been compared [16], saccade activations overlapped reaching activations in premotor and parietal regions. Similar results have been obtained when focusing on the planning stage of both hand and eye movements [17]. These and many other works [18–24] support the idea that the cortical representations for diverse movements, specifically frontal and parietal circuits for limb and eye movements, are highly distributed and overlapping in the human brain [25]. This overlap, which can be surprising at first, maybe is not so surprising if one takes into account that reaching and saccade movements are naturally coupled in daily life, and, from the point of view of efficient coding, it would not make much sense to have separate machinery to code similar planned movements that only differ in the effector used to execute them [24]. Thus, it would not be unreasonable to think that eye movements could be used in some way to generate brain activity related with limb movements.

Departing from the notion of this coupling between arm and eye, and from previous rehabilitation systems in which participants control virtual elements through their limb movements [26,27], the aim of this paper is to study a new approach to activate the sensorimotor system that does not require limb movements. The essence of the new idea is to change the traditional effector, by using the eyes instead of the limbs to control objects. In the present work, the object is a virtual paddle that is controlled by the participants in the context of a digital game. In three different conditions of the experiment, participants move the virtual paddle to hit a ball using their hand (with a hand tracking system), using their eyes (with an eye tracking system) or are merely observers of the virtual game (baseline). One would naturally expect activations in frontoparietal motor regions when comparing the traditional way of control via hand movements with the baseline. More interestingly, a relatively similar frontoparietal activity might be expected when participants are using the eye as effector due to the overlapping of brain circuits for limb and eye movements. Furthermore, if this expectation is confirmed, it could open a new potential application in the field of neurorehabilitation.

## Materials and Methods

### Subjects

18 right-handed [28] neurologically healthy subjects (11 female, 7 male) between 19 and 36 years of age (mean = 20.8; SD = 3.8). They had normal or corrected-to-normal vision. They gave their written informed consent. The study was approved by the local Ethics Committee (University of La Laguna) and was conducted in accordance with the Declaration of Helsinki. The individual in this manuscript has given written informed consent (as outlined in PLOS consent form) to publish these case details.

### Task

**Fig 1:**
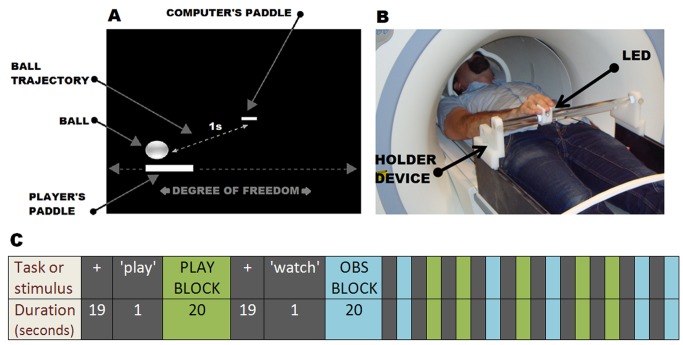
Experimental setup. (A) The digital game. The participants controlled a paddle to hit an approaching ball. The display has a 3D feel, so the more distant computer’s paddle is smaller and further away. In the first run they controlled the paddle using an fMRI-compatible eye tracking system; in the second run, the paddle was controlled by moving one LED that slid over a custom support (B). (C) Schematic representation of a run. The lower row shows the duration of the task or stimulus represented in the upper row. Each run consisted of 6 observation blocks (OBS BLOCK; represented in blue) and 6 execution blocks (PLAY BLOCK; represented in green). Execution and observation blocks were preceded by a fixation task where participants stared at a grey cross in the middle of a black screen for 19s (fixation condition; represented by the + sign). After each fixation task, an advisory word (“play” or “watch”) appeared for 1s to inform the subject about the next phase.

A virtual environment, using Visual C# and DirectX, was developed in previous research, where the subjects play a paddle and ball game from an egocentric perspective [29]. It was based on an earlier video tennis game. Participants had to prevent the ball entering the space behind them by trying to hit the approaching ball back towards the opponent (the computer), who controlled its own paddle to hit the ball in a similar way (Fig. 1A). The paddle had one degree of freedom (left-right) and its shape was cuboid. The time it took for the ball to complete a trajectory was 1 sec. The rebound angle was unpredictable for the participants so they could not anticipate the ball’s trajectories.

The experiment consisted of two different parts. Each one corresponded to one MRI run. The difference between both parts was the way the participants controlled their paddle during the game: with their eyes or with their hand. In the first part of the experiment (run1), the participants used their gaze to control the virtual paddle. This was done by using an MRI-compatible eye tracking system (MReyetracking, Resonance Technology Company, Northridge, CA), which tracks the participant’s gaze point in real-time (i.e., where they are looking). This system is supported by the ViewPoint EyeTracker software (Arrington Research, Inc., Scottsdale, AZ) and includes a Software Developer’s Kit (SDK) that allows programs like the virtual game to seamlessly interface with the eye tracker. Using this SDK, the gaze point horizontal coordinates were transformed into positions of the virtual paddle, which allowed the participant to control it in real-time. Eye movements had been previously smoothed by the eye tracking software using a simple moving average algorithm of 10 points to reduce noise. The logs were recorded unfiltered for further analysis.

In the second part of the experiment (run2), the participants used an fMRI-compatible hand tracking system to move the virtual paddle. Such a system is based on the Nintendo Wii Remote and allows, in real time, a more natural control of an object than a mouse, as has been previously published by our group [30]. It works by introducing one LED in the magnet room, and by receiving its signal with a Wii Remote located in the control room. The subjects controlled the paddle moving the LED that slid over a custom support (Fig. 1B). It was decided to perform this hand controlled part in second place to avoid any influence in the eye controlled part.

Both runs consisted of three conditions: execution, observation and fixation. The execution condition consisted of six 20 s blocks where the participant was playing against the computer. The players had 10 attempts to hit the ball during each game (in this paper, a game will mean a 20 s block). The participants only observed another six games during the observation condition. A replay of a previous game was considered for use here, but it was decided not to do it that way to prevent neural repetition suppression, that is, repeated stimulus processing is often associated with a reduction in neural activity [31], which could enhance the differences in activity between the observed and the executed conditions. The observed games were similar to the executed games, but in these cases the two paddles were controlled by the computer. All the observed games were different as they used different ball trajectories for every block and for the two runs. The two paddles were always visible during the execution and observation periods. Execution and observation blocks were presented in random order and preceded by a fixation task where the player stared at a grey cross in the middle of a black screen for 19s (fixation condition), as can be seen in Fig. 1C. An advisory word (“play” or “watch”) then appeared for 1s to inform the subject about the next phase. Eye movement data and number of hits and errors on every game were recorded for further analysis during the two runs.

### Procedure

The subjects were told that they would play a game inside the MRI scanner where their aim was to hit a ball as many times as they could. They were told that during the first part of the experiment they would use their gaze to play and in the second part they would use their hand. They were asked to move their head and trunk as little as possible. Visual stimuli were given via MRI compatible eyeglasses (Visuastim, Resonance Technology, Northridge, CA). The eyeglasses had a resolution of 800×600 pixels, 32 bit color depth and a refreshing rate of 60 Hz. The angle of vision corresponded to 30×22.5°. No audio stimuli were delivered. The eye tracking system was calibrated following the instructions of the user manual for each participant. The participants then had a practice session inside the MRI scanner to ensure that they could control the paddle with the gaze properly, where they had 200 attempts to hit the ball, which is four times more than the 50 proposed as the minimum to learn a new one-dimensional visuomotor mapping [32]. After the first run they had another practice session but this time they controlled the paddle with the hand. The participants were instructed to look at the paddle during the observation conditions in the same way they did in the execution conditions. No individual calibration was required for the hand tracking system.

### MR image acquisition

Axially oriented functional images were obtained by a 3T Signa HD MR scanner (GE Healthcare, Waukesha, WI, USA) using an echo-planar-imaging gradient-echo sequence and an 8 channel head coil (TR = 2000 msec, TE = 22 msec, flip angle = 75°, matrix size = 64 x 64 pixels, 36 slices, 4 x 4 mm in plane resolution, spacing between slices = 4 mm, slice thickness = 3.3 mm, interleaved acquisition). The head was stabilized with foam pads. The slices were aligned to the anterior commissure—posterior commissure line and covered the whole brain. Functional scanning was preceded by 18 seconds of dummy scans to ensure tissue steady-state magnetization. 241 volumes were taken during each of the two runs for every participant. High resolution sagittally oriented anatomical images were also collected for anatomical reference. A 3D fast spoiled-gradient-recalled pulse sequence was obtained (TR = 6 msec, TE = 1 msec, flip angle = 12°, matrix size = 256 x 256 pixels, .98 x .98 mm in plane resolution, spacing between slices = 1 mm, slice thickness = 1 mm).

### Image pre-processing and analysis

Data were checked for artifacts and then analyzed using SPM8 (www.fil.ion.ucl.ac.uk/spm/). The images were realigned, unwarped and normalized to the MNI space. Normalization success was validated by visual inspection. The normalized images of 2 x 2 x 2 mm were smoothed by a full width at half maximum (FWHM) 8 x 8 x 8 Gaussian kernel. Data were monitored for motion artifact using an artifact detection toolbox (www.nitrc.org/projects/artifact_detect/). A timepoint which deviated from the previous one by more than 4 SD, 1 mm, or .01 degrees was marked as an outlier timepoint. The 18 subjects included in the analyses had less than 5% of outliers. A block design in the context of a general linear model was implemented for individual subject analyses. Two sessions (one for the first run and one for the second run) with three regressors each one were created by modeling the BOLD response to each stimulus condition (i.e., execution, observation and fixation). Thus, six regressors were included in the design matrix: *eye play*, *observation1*, *fixation1*, *hand play*, *observation2*, and *fixation2*. The conditions were modeled using a box-car function convolved with the HRF. Apart from these conditions, outlier timepoints and realignment parameters were included as nuisance regressors. Activation maps were generated for each subject by applying t statistics. Three contrasts of interest were computed in the first level: *eye play* > *observation1* (for reasons of simplification here it will be called EYE_PLAY > OBS), *hand play* > *observation2* (HAND_PLAY > OBS) and *hand play* > *eye play* (HAND_PLAY > EYE_PLAY). These first level contrast images were taken to the second level (group analysis) for a random effects analysis, were its symmetric contrasts were also computed. Statistical maps were set at a voxel-level threshold of p<0.05, FDR corrected, k = 25. Anatomical locations and Brodmann's areas were determined using xjView 8.12 (www.alivelearn.net/xjview8/). After the group analysis, a conjunction analysis using the Minimum Statistic compared to the Conjunction Null method [33] was performed to determine voxels that were activated by the two modalities of game. Statistical activations for the group EYE_PLAY > OBS and HAND_PLAY > OBS contrasts were individually thresholded, binarized, and multiplied, resulting in a conjunction map revealing brain areas activated by both playing with the eye and the hand.

### Behavioral analysis

Eye tracking records provided normalized X and Y coordinates of the direction-of-gaze with respect to the game window coordinate system, with a temporal resolution of 30Hz. For example, (0.0, 0.0) means that the position of gaze was in the top left hand corner of the game window and (1.0, 1.0) means that the position of gaze was in the bottom right hand corner. Spatial resolution within the coordinate system was 640 x 480.

Quantitative analyses using different methods were performed. As a first approach, eye tracking data were imported to OGAMA software [34] for event detection and for preparation of statistical analysis. Detection of fixations was based on the dispersion-threshold-identification algorithm [35], with a dispersion radius of 1° and a minimum fixation length of 100 ms. Gaps between the fixations were classified as saccades. The following parameters were then computed for each subject and experimental condition: average fixation duration, average fixation count, and average saccade length.

For a better understanding of the eye scanning strategies, the records of the eye movements were processed and plotted with the help of customized software based on Matlab. Eye displacement, for each block, was calculated as the addition of the Euclidean distances between the gaze position of each sample and the gaze position of the following sample. After this, total eye displacement was calculated for each condition *(eye play*, *observation1*, *hand play*, *and observation2)* by adding the eye displacements of its corresponding blocks. As a measure of performance, the percentage of hits was calculated for the *hand play* and the *eye play* conditions.

When looking at the plots it was found that participants mainly used two basic strategies to hit the ball (S1 Fig.). 1: *Saccade strategy*: Participant executed an initial saccade to approximate to the estimated collision point, and waited for the collision (then using small corrections if necessary). 2: *Pursuit strategy*: The participant (horizontally) pursued the ball to try to hit it in a final collision point. This strategy was the most frequent one, as is explained in the results section. For every executed or observed trial, the customized software classified the corresponding strategy in *saccade* (the horizontal eye velocity exceeded 60°/s during the approach of the ball) or *pursuit* (the horizontal eye velocity did not exceed 60°/s during the approach of the ball). Visual inspection was used to verify that the algorithm outcome was adequate to the authors’ criteria in more than 95% of the tested trials. The percentage of *pursuit strategy* used in every condition was calculated and selected as a measure of the strategy used.

## Results

### Neural

**Fig 2:**
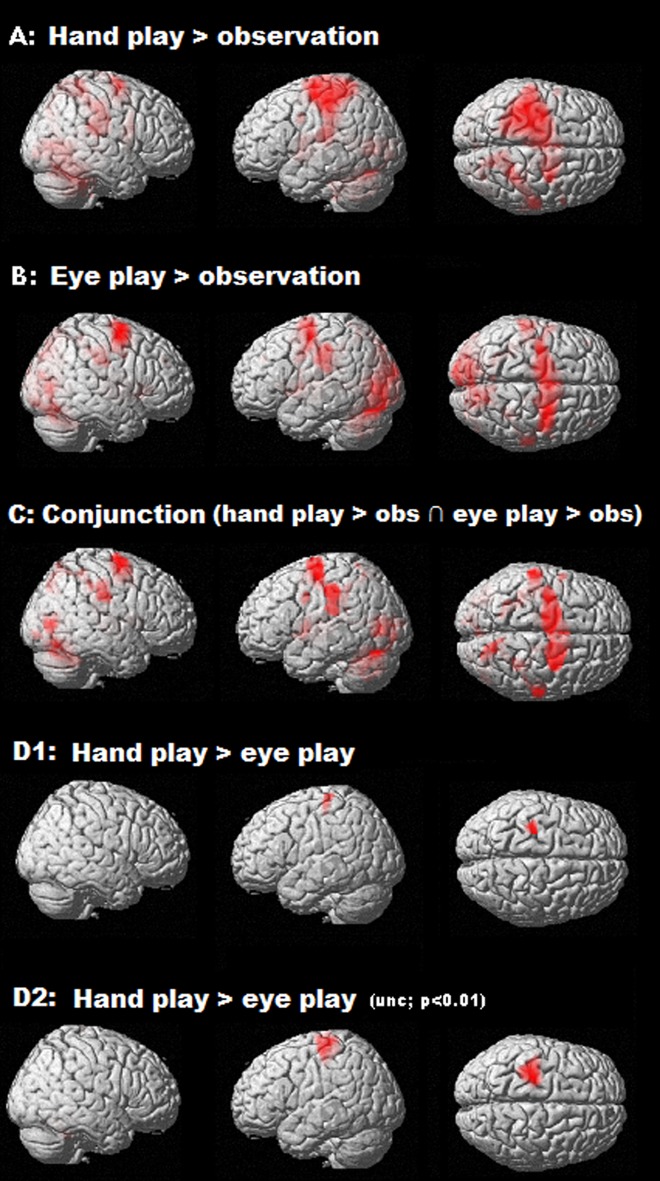
Results of the main contrasts. (A, B, C, D1). Threshold = p<0.05 at the voxel level, FDR; k = 25. The lack of differences in activity between the HAND_PLAY and the EYE_PLAY condition remained in most of the brain regions when a much less conservative threshold was used (D2). Threshold: p<0.01 a at the voxel level, uncorrected; k = 1.

Fig. 2A and Table 1 summarize the increases in the BOLD signal in the HAND_PLAY > OBS contrast. This comparison showed an extensive network, centred on the left primary motor and primary somatosensory cortex, which was mainly made up of (left) fronto-parietal regions, and, to a lesser extent, some temporal, and occipital regions. Sub-lobar, cerebellar and midbrain regions were also activated. As regards its symmetric contrast, no significant BOLD signal increase was detected in the OBS > HAND_PLAY comparison.

**Table 1:** Summary of the main fMRI results.

Region	BA	*Voxels*	*[X Y Z]*	*Z score*
**hand play > observation**				
L Postcentral gyrus, L precentral gyrus; L,R: cerebellum, temporal lobe, parietal lobe, occipital lobe, sub-lobar, midbrain	6,40,7,3,2,4,1,18,19	55412	[−30 −30 50]	6.19
R Insula, R putamen		1357	[38 2 16]	3.85
L precentral gyrus	6,9	147	[−56 2 36]	4.24
**hand play < observation**				
No significant activations				
**eye play > observation**				
L,R Precentral gyrus; L,R: parietal lobe, occipital lobe, sub-lobar, midbrain, cerebellum, temporal lobe; L,R: Postcentral gyrus	6,18,19,7,40,4,2,3,1	52454	[−28 −10 50]	6.86
L Insula		359	[−46 −2 6]	4.21
R: Supplementary motor area, median cingulate and paracingulate gyri	24,31	272	[14 −18 44]	3.85
**eye play < observation**				
No significant activations				
**conjunction (hand play > obs. ∩ eye play > obs.)**				
L,R Precentral gyrus; L,R: parietal lobe, occipital lobe, sub-lobar, midbrain, cerebellum, temporal lobe; L,R: Postcentral gyrus	6,18,19,7,40,4,2,3,1	28062	[−28 −10 50]	
L Insula		257	[−46 −2 6]	
R putamen		246	[30 4 2]	
L precentral gyrus	6,9	114	[−56 2 36]	
R: Supplementary motor area, median cingulate and paracingulate gyri	24,31	237	[14 −18 44]	
**hand play > eye play**				
L Precentral gyrus, L postcentral gyrus	6,1,3,4	312	[−30 −24 62]	4.57
hand play > eye play*				
L Precentral gyrus, L postcentral gyrus	6,1,3,4,2,40	2371	[−30 −24 62]	4.57
R Temporal lobe, R sub-gyral	37,36	174	[40 −38 −12]	2.79
R Cerebellum		1447	[6 −62 −12]	3.49
R Cerebellum		119	[2 −44 −32]	2.79
L,R: Anterior Cingulate	32	28	[0 36 −10]	2.26
R Sub-lobar		92	[22 −42 14]	2.14

Anatomical structures and Brodmann Areas (BA) are reported with corresponding MNI coordinates of peak activity in each cluster. Threshold: p<0.05 at the voxel level, FDR corrected for multiple comparisons, k = 25 (with the exception of *: p<0.01 at the voxel level, uncorrected, k = 1). Local maxima are at least 4.0 mm apart. L/R: left and right hemispheres.

The results of the EYE_PLAY > OBS contrast are shown in Fig. 2B and Table 1, where many of the activated voxels are located in areas related with motor aspects [36]. It is noteworthy the extended, bilateral activity found in a region centred in Brodmann area 6 (premotor cortex, supplementary motor area, and, to a lesser extent, a part of the primary motor cortex) which includes oculomotor regions like the frontal and the supplementary eye field [15]. Bilateral activity was also found in several regions of the inferior and superior parietal lobules (as the supramarginal gyrus, the angular gyrus and the precuneus). The occipital lobe and the cerebellum were also bilaterally activated, as well as midbrain and sublobar regions, and, to a lesser extent, some temporal areas as Fig. 2B shows. As regards its symmetric contrast, no significant BOLD signal increase was detected in the OBS > EYE_PLAY comparison.

The conjunction analysis between the above mentioned contrasts (EYE_PLAY > OBS and HAND_PLAY > OBS) revealed the brain activations shared by the two modalities of game (Fig. 2C, Table 1). These regions were fairly similar to those activated in the EYE_PLAY > OBS contrast (premotor cortex, supplementary motor area, a part of the primary motor cortex, parietal, occipital, cerebellum, midbrain, sublobar, and, to a lesser extent, some temporal areas).

The last contrast of interest, HAND_PLAY > EYE_PLAY is shown in Fig. 2D1. This comparison showed just one cluster located in the left primary motor and primary somatosensory cortex. This lack of differences in activity between the HAND_PLAY and the EYE_PLAY condition remained in most of the brain regions when a much less conservative threshold was used: Fig. 2D2 presents the result of the same contrasts but using p<0.01, uncorrected. Here one can see an extension of the cluster centered in the left primary motor cortex, and also some other activity, mainly in cerebellar regions (Table 1). As regards the symmetric contrast, no significant BOLD signal increase was detected in the EYE_PLAY > HAND_PLAY comparison.

**Fig 3:**
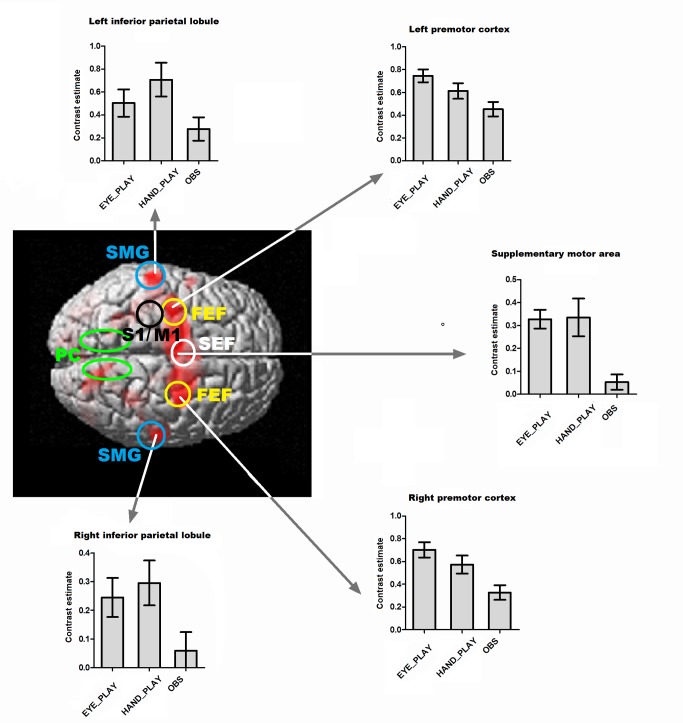
Contrast estimates in frontoparietal regions. The figure shows contrast estimates for each of the EYE_PLAY, HAND_PLAY and OBS conditions (against fixation condition) in five representative voxels (superimposed on the conjunction analysis image): left inferior parietal lobule [−56 −28 46]; left premotor cortex [−28 −10 50]; supplementary motor area [−10 2 52]; right premotor cortex [28 −2 60]; right inferior parietal lobule [60 −24 40]. Error bars depict the standard error. In all cases, activations were statistically significantly higher in both EYE_PLAY and HAND_PLAY conditions when compared with the OBS condition. No significant difference was observed between EYE_PLAY and HAND_PLAY conditions. Circles and ellipses indicate locations of frontoparietal areas mentioned in the present study: FEF = frontal eye fields; SEF = supplementary eye field; S1/M1 = left primary somatosensory and primary motor cortex (arm/hand motor area); PC = precuneus; SMG = supramarginal gyrus.

Fig. 3 shows contrast estimates (beta values) for each of the EYE_PLAY > FIXATION, HAND_PLAY > FIXATION and OBS > FIXATION contrasts in five representative voxels. The contrasts were calculated as explained in the Materials and Methods section. The representative voxels were chosen because they were points of local maxima activity during the eye play condition and also because they were located in frontoparietal regions. These areas were as follows: left premotor cortex [−28 −10 50]; right premotor cortex [28 −2 60]; supplementary motor area [−10 2 52]; left inferior parietal lobule [−56 −28 46]; right inferior parietal lobule [60 −24 40].

### Behavioral

Data from two of the participants got lost due to technical problems in the computer that recorded them, thus the behavioral analysis was conducted using an N = 16. Three paired-samples t-test were performed for each eye parameter (fixation duration, fixation count, saccade length, eye displacement and percentage of *pursuit strategy*): one for the *eye play-observation1* pair, one for the *hand play-observation2* pair, and one for the *eye play-hand play* pair. These comparisons were chosen because they correspond with the image contrasts presented above. None of the eye parameter comparisons revealed a significant difference (p = 0.05) even before correcting for multiple comparisons (with the exception of the *hand play-observation2* comparison for the percentage of *pursuit strategy*, although this result was non-significant when applying a Bonferroni correction). The results of the comparisons and descriptive statistics are shown in S1 and S2 Tables. Furthermore, using the OGAMA Attention Map Module, group attention maps were calculated as aggregated Gaussian distributions of each fixation in an experimental condition [34]. The results show similar maps for all the conditions (S1 Fig.). Taken together, the results indicate that eye scanning strategies were fairly similar in each executed task and its corresponding control condition and also in the two executed tasks.

As regards the performance, the percentage of hits was high in both execution conditions (above the 90%), though it was significantly higher for the *hand play* (M = 97.8, SD = 4.34) than for the *eye play* condition (M = 90.0, SD = 13.80), t(15) = −2.22, p = .042, d = 0.54, as a paired-samples t-test showed.

### Regression analysis

Two SPM regression analyses were conducted (N = 16) to look for relationships between the activations of the EYE_PLAY > OBS contrast and variations in the behavioral parameters. In order to do this, the first level EYE_PLAY > OBS contrast images and several covariate values were included in two different design matrices. In the first model, covariates corresponded to the five eye parameters and the % of hits for the EYE_PLAY condition. In an additional model, covariates were calculated as the difference in each eye parameter between the *eye play* and the *observation1* condition. For both models, results did not show any positive or negative relationship between any of the covariates and the activations of the EYE_PLAY > OBS contrast (p<0.05, FDR corrected, k = 25). Furthermore, when regressing out the covariates, results were similar to those previously obtained with the 18 participants and no behavioral covariates (see example S2 Fig., obtained from the second model).

## Discussion

This work studies the brain activity of healthy volunteers who controlled a virtual object in the context of a digital game by applying two different methods: using their right hand or using their gaze. The results show extended activations, many of these in sensorimotor areas, not only when participants played in a traditional way (using their hand) but also when they used their gaze to control the virtual object. The implications of these findings are described below.

### Using gaze to control a virtual object increases the activity in premotor and parietal cortex

The contrast HAND_PLAY > OBS shows different activations, especially in left sensorimotor regions that can be clearly related with the movements of the right limb during the *hand play* condition (this outstanding motor activity was to be expected because the participants were moving their arm during the *hand play* condition, but not during the observation condition). But what is more interesting for the present research is the result of the contrast EYE_PLAY > OBS, that shows widespread activations in the premotor cortex (extended to some primary motor regions), as well as in the parietal cortex, the brainstem and the cerebellum. Such activity can be related with several cognitive and motor processes as explained below.

In comparison with the non-interactive *observation* condition, the interactive *eye play* condition requires more careful control and execution of the eye movements (though eye movements are present in both cases). This fact could have been responsible for some of the increase in activity during the *eye play* condition in areas that are involved in the control of the ocular movements [15], like the frontal eye fields (around the lateral part of the precentral sulcus), the supplementary eye field (in the supplementary motor area), the parietal eye fields (adjacent to the supramarginal and the angular gyri), and the cerebellum. Areas involved in the execution of ocular movements, like the superior colliculus in the midbrain were also activated [37,38]. On the other hand, to help minimize differences in eye movements with respect to the baseline, the participants were instructed to look at the paddle during the observation conditions in the same way they did in the execution conditions. Such minimization must have happened up to some point if one looks at the behavioral results, which did not show significant differences between both conditions in the different eye parameters examined. Furthermore, the differences in activity in the EYE_PLAY > OBS contrast does not appear to be related with differences in eye parameters or performance, as indicated by the regression analysis. Thus, it does not seem that the increases of activity found here are mainly due to differences in ocular movements.

Ocular movements are closely related with attentional processes [39]. Indeed, such processes should activate similar regions, according to several lines of evidence which suggest that eye movements and visuospatial attention share overlapping brain mechanisms [40]. For example, it has been shown that, while fixating the gaze, attentive vs passive tracking of moving targets activates premotor and parietal areas (as well as occipitotemporal regions) related with the visual processing of motion [41]. Besides which, it has been proposed that spatial attention results from an activation of the same circuits that program eye movements (and also other motor activities like reaching and grasping), which has been called the *premotor theory of attention* [42], and was developed with the intention of having a unitary explanation of a variety of visual-related attentional phenomena. To date, the exact relationship between eye-motor and attentional mechanism is still a matter of research, and such processes are difficult to separate, even more so using situations with high ecological validity like the one presented here. In any case, it is very likely that attentional effort was higher during the execution than during the observation conditions, and this could be responsible for part of the increase in activity found in the EYE_PLAY > OBS contrast [43]. Activations in the occipital cortex are also probably related with attentional processes [44].

It is important to note that the ocular movements performed during the *eye play* condition are not only pursuit or saccade movements towards spatial positions, in the sense that they have been used to control an object (via eye tracking). This means that during the *eye play* condition, participants performed a complex visuomotor task, similar to other everyday tasks (like controlling a mouse cursor), but using a different effector (the eye). This increase of visuomotor complexity in the task (from the passive observation condition to the active eye play condition) probably led to activations in several visual and motor regions (see Fig. 2B). Differences in activity between both tasks can also be seen in Fig. 3: in both conditions participants were moving their eyes, but the values of the parameter estimates were higher in the eye play condition.

To summarize, when compared with an observation task (with a similar visual input), using gaze to control a virtual object is associated with an increase of activity in motor regions that can be related with motor processes, and also with cognitive processes. The present work has not been designed to discriminate the different factors that can modulate the activity found. This would be a methodological challenge [13] which could perhaps be addressed in further research using simpler tasks. The important point here has been finding extended activations in motor regions, which is in line with our expectations.

### Similar activations in eye control and hand control modalities

Another important finding of this study comes from the combination of two of the obtained results. On the one hand, the conjunction analysis showed motor activations (especially in the premotor cortex). On the other hand, the outcome of the HAND_PLAY > EYE_PLAY contrast showed activation in the left primary motor cortex. This activation was to be expected because (only) during the *hand play* condition were the participants moving their right arm, but the lack of significant differences in the rest of the brain, even when using a less conservative threshold, is also of interest (Fig. 2D2). Taken together, these results indicate that, with the exception of the primary motor cortex, regional motor activity was similar regardless of what the effector was: the arm or the eye. Here it should be noted that although similar regions are activated using the two effectors, it is very likely that different populations of neurons are involved in each control-process. Some neurons in the same voxel (or across nearby voxels blurred in the smoothing process) could be involved in multi-motor control (responding both during eye and hand movements), while other neurons could be effector–specific (some neurons could respond during eye movements and some neurons could respond during hand movements). Thus, the resolution of the fMRI data limits our capacity to understand the overlaps between eye control and hand control activity. The combination of different techniques and approaches to investigate function, structure, and intrinsic connectivity [25] can provide new insights into the functional specificity of the networks involved in the control of a virtual object. In any case, the results presented here are supported by previous functional neuroimaging studies that report widespread, overlapping mechanisms for hand and eye movements [16]. This overlap may not be so evident in the monkey brain, according to works that have identified different effector-related regions in the posterior parietal cortex [45–48]. However, it has been pointed out that such effector selectivity is actually limited: each region contains neurons selective for the non-preferred effector (eye or arm), and many neurons in these regions respond for both effectors [24,49]. The contrast estimates shown in Fig. 3 also support the idea that controlling the paddle with gaze produces activity in the motor system in a very similar way to that produced when playing the game in a more usual way, i.e., when controlling the paddle with the hand.

The potential application of these findings is explained below.

### The obtained results can be of interest in the field of neurorehabilitation

A key factor for the reorganization of neural functions is the stimulation of the damaged networks [50], and the method presented in this paper can be useful to reach this goal, providing advantages and improvements to the others available in this moment of time. In first place, it has been shown that playing the game with the gaze produces more activity than an observation task with a similar visual input. Thus, adding an active control component via eye movements might be useful to increase the benefits of previous action observation therapies. Another reason these findings can be useful for neurorehabilitation is that the brain activations were fairly similar when playing with the hand or with the eye, suggesting that different motor regions could benefit by using this approach. Also of interest is the fact that the participants did not have previous experience with the game in any of the two modalities of the control of the paddle. The importance of this is that the patients that could take benefit from this approach would not need to have previous experience with the specific eye tracking rehabilitation system used to perform the therapy. Simply by directly controlling virtual objects (or even virtual limbs) with the eyes, activation in motor regions is expected to happen, without needing a specific manual training, a training that many times is not possible due the motor dysfunctions. The necessity of the presence of the therapist could also be reduced, and (unlike what happens in motor imagery) it is easy to check the execution of the patient and one can obtain registers of the eye movements. Another advantage of this approach is that the control of a virtual object with the gaze may be more attractive for the patients than other kinds of tasks used in rehabilitation, which therefore may help the patient to adhere to the therapy. For example, motor imagery can be difficult for patients to perform, or, in action observation therapies, paying attention to the stimuli during long periods could be boring for the patients. But the control of a virtual object with the gaze can even be rewarding. In fact, controlling items within an interactive environment is the base of most of the digital games that are played just for fun. In the future, attractive neurorehabilitation gaze-based systems could be developed for the users, which may be especially useful in the case of children and adolescents, populations that can also be affected by cerebrovascular accidents despite their youth [51,52].

For all the aforementioned reasons, and taking into account that eye tracking can be a non-expensive technique [53,54], it would be worthwhile continuing this line of research, by performing further experiments on clinical populations, whose results could be applied to the development of new systems for the rehabilitation of patients with motor deficits.

### Conclusions

When compared with a passive observation task (including eye movements), the active control of virtual elements with the eye enhances brain activity in sensorimotor regions. In many of these regions, the activations are fairly similar to those obtained when the virtual elements are controlled with a more usual effector: the arm. To the best of our knowledge, this is the first work studying brain activity related with the eye control of virtual elements, which could be a promising approach to enhance motor activity without resorting to limb movements. The results presented here may be of interest to researchers, developers and medical professionals working in the field of the neurorehabilitation.

## Supporting Information

S1 Fig Game strategies and attention maps. (Top) Sample of the two basic strategies used to hit the ball. Traces represent the time course of gaze (blue) and ball (red) horizontal position of one participant during three consecutive trials. The vertical continuous lines mark the beginning of each trial. The vertical dotted lines mark the moment in which the participant’s paddle hit the ball. Strategy was classified, for every trial, as *saccade strategy* or *pursuit strategy*. (Bottom) Group attention maps calculated as aggregated Gaussian distributions of each fixation in an experimental condition. The results show similar maps for all the conditions. Most of the fixations were performed around a central position, possible because this is the optimal position to wait for the next ball and also for the natural tendency of the eyes to return to the primary eye position after a displacement.
(TIF)

S2 Fig Regression analysis. Activations in the EYE_PLAY > OBS contrast, when regressing out covariates related activity. Covariates were calculated as the difference in each eye parameter (fixation count, fixation duration, saccade length, total eye displacement, % of *pursuit strategy*) between the *eye play* and the *observation1* condition. N = 16. Note that these results are similar to those obtained with N = 18 and no behavioral covariates (see Fig. 2B). Threshold: p<0.05 at the voxel level, FDR; k = 25.
(TIF)

S1 Table Eye parameter descriptive stastistics. Means and SD of the different eye parameters in the four conditions of the experiment.
(DOC)

S2 Table Eye parameter comparisons. Results of the paired samples t-tests for the different eye parameters. For each eye parameter, three comparisons were performed. P-values are not corrected for multiple comparisons.
(DOC)

## References

[1] Szameitat AJ, Shen S, Conforto A, Sterr A. Cortical activation during executed, imagined, observed, and passive wrist movements in healthy volunteers and stroke patients. Neuroimage. 2012;62: 266–280. 10.1016/j.neuroimage.2012.05.009 22584231

[2] Lemon RN, Porter R. Afferent input to movement-related precentral neurons in conscious monkeys. Proc R Soc Lond B Biol Sci. 1976;194: 313–339. 11491 10.1098/rspb.1976.0082

[3] Dechaumont-Palacin S, Marque P, De Boissezon X, Castel-Lacanal E, Carel C, Berry I, et al Neural correlates of proprioceptive integration in the contralesional hemisphere of very impaired patients shortly after a subcortical stroke: An fMRI study. Neurorehabil Neural Repair. 2008;22: 154–165. 17916656 10.1177/1545968307307118

[4] Decety J. Do imagined and executed actions share the same neural substrate? Brain Res Cogn Brain Res. 1996;3: 87–93. 8713549 10.1016/0926-6410(95)00033-x

[5] Zimmermann-Schlatter A, Schuster C, Puhan MA, Siekierka E, Steurer J. Efficacy of motor imagery in post-stroke rehabilitation: a systematic review. J Neuroeng Rehabil. 2008;5: 8 10.1186/1743-0003-5-8 18341687 PMC2279137

[6] Cattaneo L, Rizzolatti G. The mirror neuron system. Arch Neurol. 2009;66: 557–560. 10.1001/archneurol.2009.41 19433654

[7] Plata Bello J, Modroño C, Marcano F, Gonzalez-Mora JL. Observation of simple intransitive actions: the effect of familiarity. PLOS ONE. 2013;8(9): e74485 10.1371/journal.pone.0074485 24073213 PMC3779225

[8] Plata Bello J, Modroño C, Marcano F, Gonzalez-Mora JL. The mirror neuron system and motor dexterity: What happens? Neuroscience. 2014;275: 285–295. 10.1016/j.neuroscience.2014.06.010 24952330

[9] Buccino G, Solodkin A, Small SL. Functions of the mirror neuron system: Implications for neurorehabilitation. Cogn Behav Neurol. 2006;19: 55–63. 16633020 10.1097/00146965-200603000-00007

[10] Garrison KA, Winstein CJ, Aziz-Zadeh L. The mirror neuron system: a neural substrate for methods in stroke rehabilitation. Neurorehabil Neural Repair. 2010;24: 404–412. 10.1177/1545968309354536 20207851 PMC11692383

[11] Sale P, Franceschini M. Action observation and mirror neuron network: a tool for motor stroke rehabilitation. Eur J Phys Rehabil Med. 2012;48: 313–318. 22522432

[12] Ertelt D, Small S, Solodkin A, Dettmers C, McNamara A, Binkofski F, et al Action observation has a positive impact on rehabilitation of motor deficits after stroke. Neuroimage. 2007;36: T164–T173. 17499164 10.1016/j.neuroimage.2007.03.043

[13] Curtis CE. Testing animal models of human oculomotor control with neuroimaging In: Liversedge SP, Gilchrist ID, Everling S, editors. Oxford Handbook on Eye Movements. New York: Oxford University Press; 2012 pp. 383–398.

[14] Koyama M, Hasegawa I, Osada T, Adachi Y, Nakahara K, Miyashita Y. Functional magnetic resonance imaging of macaque monkeys performing visually guided saccade tasks: Comparison of cortical eye fields with humans. Neuron. 2004;41: 795–807. 15003178 10.1016/s0896-6273(04)00047-9

[15] Leigh R, Zee DS. The neurology of eye movements 4th ed. New York: Oxford University Press; 2006.

[16] Filimon F, Nelson JD, Huang RS, Sereno MI. Multiple parietal reach regions in humans: cortical representations for visual and proprioceptive feedback during on-line reaching. J Neurosci. 2009;29: 2961–2971. 10.1523/JNEUROSCI.3211-08.2009 19261891 PMC3407568

[17] Beurze SM, de Lange FP, Toni I, Medendorp WP. Spatial and effector processing in the human parietofrontal network for reaches and saccades. J Neurophysiol. 2009;101: 3053–3062. 10.1152/jn.91194.2008 19321636

[18] Beurze SM, de Lange FP, Toni I, Medendorp WP. Integration of target and effector information in the human brain during reach planning. J Neurophysiol. 2007;97: 188–199. 16928798 10.1152/jn.00456.2006

[19] Hagler DJ, Riecke L, Sereno ML. Parietal and superior frontal visuospatial maps activated by pointing and saccades. Neuroimage. 2007;35: 1562–1577. 17376706 10.1016/j.neuroimage.2007.01.033 PMC2752728

[20] Connolly JD, Goodale MA, Cant JS, Munoz DP. Effector-specific fields for motor preparation in the human frontal cortex. Neuroimage. 2007;34: 1209–1219. 17134914 10.1016/j.neuroimage.2006.10.001

[21] Medendorp WP, Goltz HC, Crawford JD, Vilis T. Integration of target and effector information in human posterior parietal cortex for the planning of action. J Neurophysiol. 2005;93: 954–962. 15356184 10.1152/jn.00725.2004

[22] Medendorp WP, Goltz HC, Vilis T, Crawford JD. Gaze-centered updating of visual space in human parietal cortex. J Neurosci. 2003;23: 6209–6214. 12867504 10.1523/JNEUROSCI.23-15-06209.2003 PMC6740538

[23] Astafiev SV, Shulman GL, Stanley CM, Snyder AZ, Van Essen DC, Corbetta M. Functional organization of human intraparietal and frontal cortex for attending, looking, and pointing. J Neurosci. 2003;23: 4689–4699. 12805308 10.1523/JNEUROSCI.23-11-04689.2003 PMC6740811

[24] Levy I, Schluppeck D, Heeger DJ, Glimcher PW. Specificity of human cortical areas for reaches and saccades. J Neurosci. 2007;27: 4687–4696. 17460081 10.1523/JNEUROSCI.0459-07.2007 PMC1876670

[25] Filimon F. Human cortical control of hand movements: parietofrontal networks for reaching, grasping, and pointing. Neuroscientist. 2010;16: 388–407. 10.1177/1073858410375468 20817917

[26] Cameirao MS, Bermudez i Badia S, Duarte Oller E, Verschure PFMJ. Neurorehabilitation using the virtual reality based Rehabilitation Gaming System: methodology, design, psychometrics, usability and validation. J Neuroeng Rehabil. 2010;7: 48 10.1186/1743-0003-7-48 20860808 PMC2949710

[27] Burstin A, Brown R. Use of a novel virtual reality system to assess and treat stroke patients with neglect—a feasibility study. Int J Rehabil Res. 2009;32: S77–S78.

[28] Oldfield RC. Assessment and analysis of handedness—Edinburgh Inventory. Neuropsychologia. 1971;9: 97–113. 5146491 10.1016/0028-3932(71)90067-4

[29] Modroño C, Navarrete G, Rodriguez-Hernandez AF, Gonzalez-Mora JL. Activation of the human mirror neuron system during the observation of the manipulation of virtual tools in the absence of a visible effector limb. Neurosci Lett. 2013;555: 220–224. 10.1016/j.neulet.2013.09.044 24080372

[30] Modroño C, Rodriguez-Hernandez AF, Marcano F, Navarrete G, Burunat E, Ferrer M, et al A low cost fMRI-compatible tracking system using the Nintendo Wii remote. J Neurosci Methods. 2011;202: 173–181. 10.1016/j.jneumeth.2011.05.014 21640136

[31] Henson RNA, Rugg MD. Neural response suppression, haemodynamic repetition effects, and behavioural priming. Neuropsychologia. 2003;41: 263–270. 12457752 10.1016/s0028-3932(02)00159-8

[32] Gozli DG, Brown LE. Agency and control for the integration of a virtual tool into the peripersonal space. Perception. 2011;40: 1309–1319. 22416589 10.1068/p7027

[33] Nichols T, Brett M, Andersson J, Wager T, Poline JB. Valid conjunction inference with the minimum statistic. Neuroimage. 2005;25: 653–660. 15808966 10.1016/j.neuroimage.2004.12.005

[34] Vosskühler A, Nordmieeer V, Kuchrvke L, Jacobs AM. OGAMA (Open Gaze and Mouse Analyzer): Open-source software designed to analyze eye and mouse movements in slideshow study designs. Behav Res Methods. 2008;40: 1150–1162. 10.3758/BRM.40.4.1150 19001407

[35] Salvucci DD, Goldberg JH. Identifying fixations and saccades in eye-tracking protocols. In: Duchowski AT, editor. Proceedings of the 2000 symposium on eye tracking research and applications. New York: ACM Press; 2000. pp. 71–78.

[36] Kandel ER, Schwartz JH, Jessell TM, Siegelbaum SA, Hudspeth AJ. Principles of Neural Science. 5th ed. New York: McGraw-Hill; 2013.

[37] Munoz DP, Fecteau JH. Vying for dominance: dynamic interactions control visual fixation and saccadic initiation in the superior colliculus. Prog Brain Res. 2002;140: 3–19. 12508579 10.1016/S0079-6123(02)40039-8

[38] Moschovakis AK. The superior colliculus and eye movement control. Curr Opin Neurobiol. 1996;6: 811–816. 9000018 10.1016/s0959-4388(96)80032-8

[39] Moore T, Fallah M. Control of eye movements and spatial attention. Proc Natl Acad Sci U S A. 2001;98: 1273–1276. 11158629 10.1073/pnas.021549498 PMC14744

[40] Grosbras MN, Laird AR, Paus T. Cortical regions involved in eye movements, shifts of attention, and gaze perception. Hum Brain Mapp. 2005;25: 140–154. 15846814 10.1002/hbm.20145 PMC6871707

[41] Culham JC, Brandt SA, Cavanagh P, Kanwisher NG, Dale AM, Tootell RB. Cortical fMRI activation produced by attentive tracking of moving targets. J Neurophysiol. 1998;80: 2657–2670. 9819271 10.1152/jn.1998.80.5.2657

[42] Rizzolatti G, Riggio L, Dascola I, Umilta C. Reorienting attention across the horizontal and vertical meridians: evidence in favor of a premotor theory of attention. Neuropsychologia. 1987;25: 31–40. 3574648 10.1016/0028-3932(87)90041-8

[43] Simon SR, Meunier M, Piettre L, Berardi AM, Segebarth CM, Boussaoud D. Spatial attention and memory versus motor preparation: Premotor cortex involvement as revealed by fMRI. J Neurophysiol. 2002;88: 2047–2057. 12364527 10.1152/jn.2002.88.4.2047

[44] Bressler DW, Silver MA. Spatial attention improves reliability of fMRI retinotopic mapping signals in occipital and parietal cortex. Neuroimage. 2010;53: 526–533. 10.1016/j.neuroimage.2010.06.063 20600961 PMC2930091

[45] Gnadt JW, Andersen RA. Memory related motor planning activity in posterior parietal cortex of macaque. Exp Brain Res. 1988;70: 216–220. 3402565 10.1007/BF00271862

[46] Platt ML, Glimcher PW. Response fields of intraparietal neurons quantified with multiple saccadic targets. Exp Brain Res. 1998;121: 65–75. 9698192 10.1007/s002210050438

[47] Snyder LH, Batista AP, Andersen RA. Coding of intention in the posterior parietal cortex. Nature. 1997;386: 167–170. 9062187 10.1038/386167a0

[48] Galletti C, Kutz DF, Gamberini M, Breveglieri R, Fattori P. Role of the medial parieto-occipital cortex in the control of reaching and grasping movements. Exp Brain Res. 2003;153: 158–170. 14517595 10.1007/s00221-003-1589-z

[49] Andersen RA, Cui H. Intention, action planning, and decision making in parietal-frontal circuits. Neuron. 2009;63: 568–583. 10.1016/j.neuron.2009.08.028 19755101

[50] Johnson-Frey SH. Stimulation through simulation? Motor imagery and functional reorganization in hemiplegic stroke patients. Brain Cogn. 2004;55: 328–331. 15177807 10.1016/j.bandc.2004.02.032

[51] Lemesle M, Manceau E, Osseby GV, Madinier-Chappart N, Moreau T, Giroud M. Stroke in children. Rev Neurol. 2001;157: 1255–1263. 11885518

[52] Arroyo HA, Tamer I. Cerebrovascular disease in childhood and adolescence. Ischemic cerebral accidents. Rev Neurol. 2002;34: 133–144. 11988908

[53] Lin YT, Lin RY, Lin YC, Lee GC. Real-time eye-gaze estimation using a low-resolution webcam. Multimed Tools Appl. 2013;65: 543–568.

[54] Shen Y, Shin HC, Sung WJ, Khim S, Kim H, Rhee PK. Evolutionary adaptive eye tracking for low-cost human computer interaction applications. J Electron Imaging. 2013;22: 16.

