# Supplementary figures and images for: Enhancing Sensorimotor Activity by Controlling Virtual Objects with Gaze
Source: PLoS One. 2015 Mar 23;10(3):e0121562. doi: 10.1371/journal.pone.0121562 (PMC4370397; doi:10.1371/journal.pone.0121562)

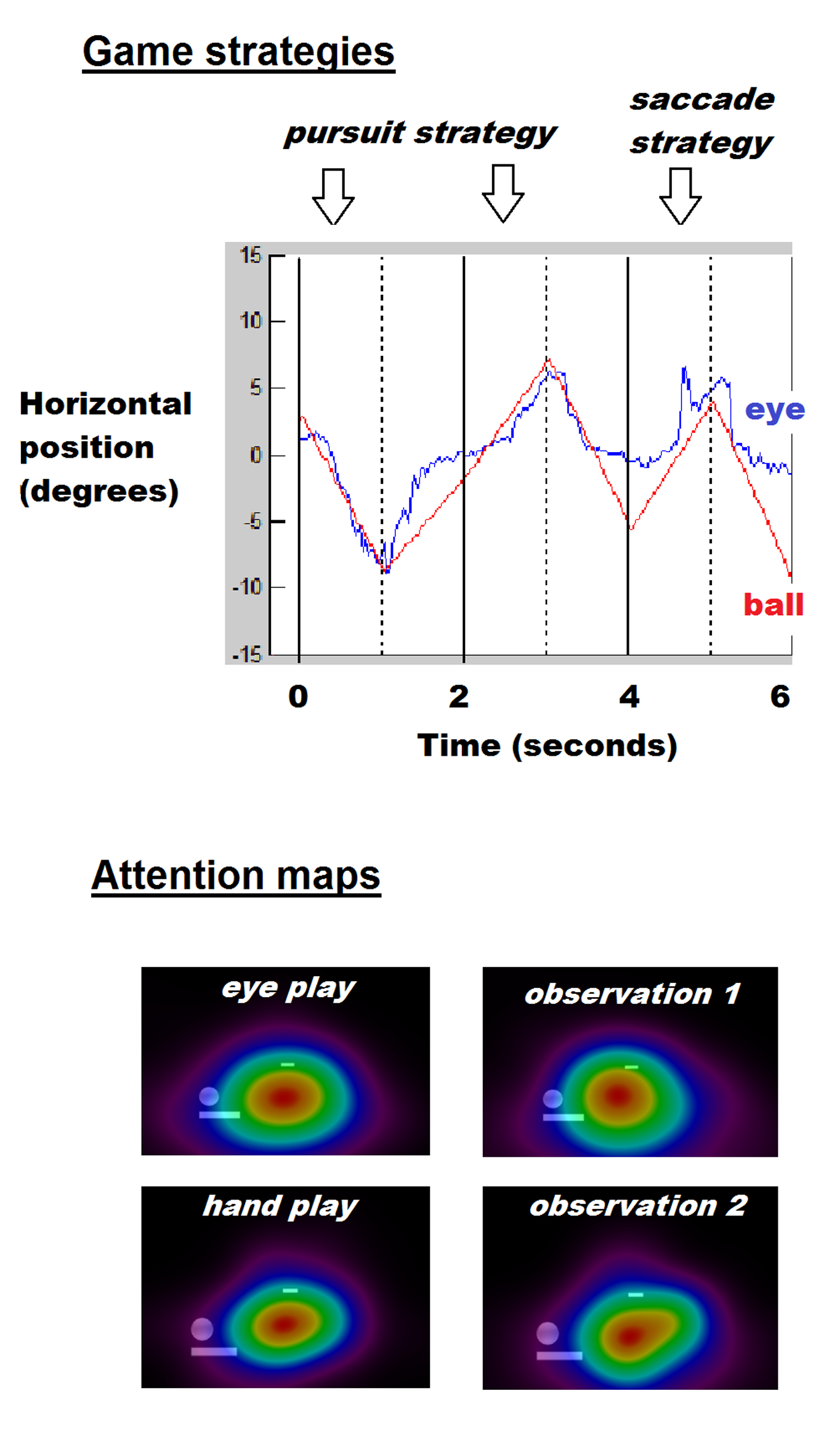

Supplement: S1 Fig — (Top) Sample of the two basic strategies used to hit the ball. Traces represent the time course of gaze (blue) and ball (red) horizontal position of one participant during three consecutive trials. The vertical continuous lines mark the beginning of each trial. The vertical dotted lines mark the moment in which the participant’s paddle hit the ball. Strategy was classified, for every trial, as saccade strategy or pursuit strategy. (Bottom) Group attention maps calculated as aggregated Gaussian distributions of each fixation in an experimental condition. The results show similar maps for all the conditions. Most of the fixations were performed around a central position, possible because this is the optimal position to wait for the next ball and also for the natural tendency of the eyes to return to the primary eye position after a displacement. (TIF) [file pone.0121562.s001.tif]

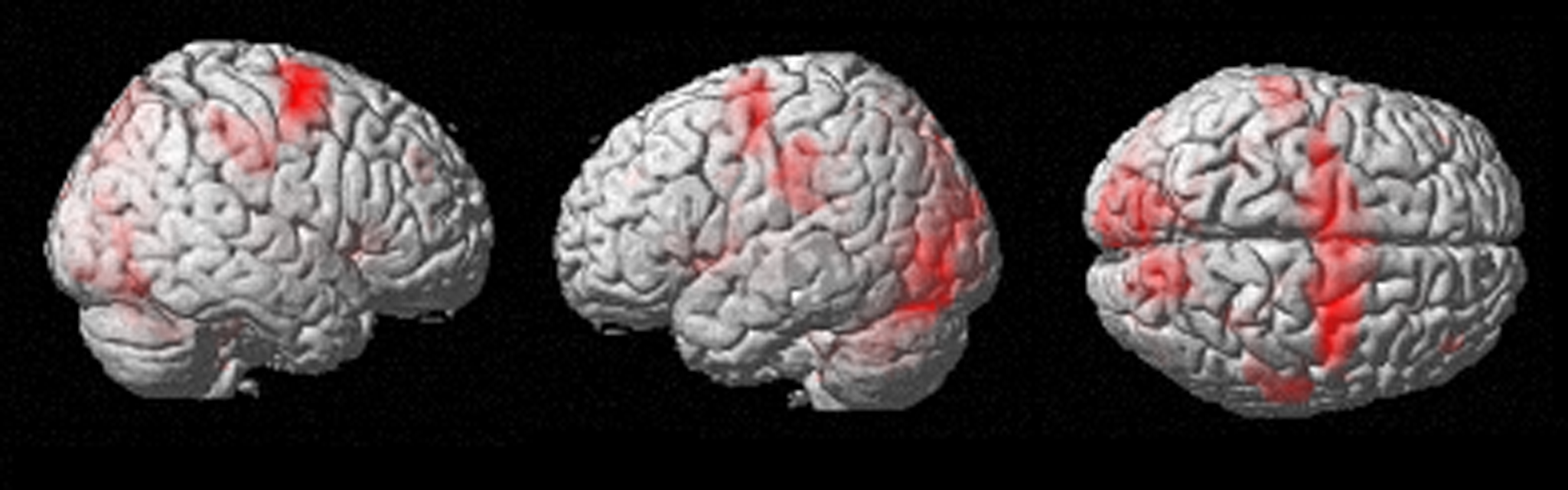

Supplement: S2 Fig — Activations in the EYE_PLAY > OBS contrast, when regressing out covariates related activity. Covariates were calculated as the difference in each eye parameter (fixation count, fixation duration, saccade length, total eye displacement, % of pursuit strategy) between the eye play and the observation1 condition. N = 16. Note that these results are similar to those obtained with N = 18 and no behavioral covariates (see Fig. 2B). Threshold: p<0.05 at the voxel level, FDR; k = 25. (TIF) [file pone.0121562.s002.tif]
