# Supplementary material for: Enhancing Sensorimotor Activity by Controlling Virtual Objects with Gaze
Source: PLoS One. 2015 Mar 23;10(3):e0121562. doi: 10.1371/journal.pone.0121562 (PMC4370397; doi:10.1371/journal.pone.0121562)
Supplement: S1 Table — Means and SD of the different eye parameters in the four conditions of the experiment. (DOC) [file pone.0121562.s003.doc]

| **Eye parameter** | **Condition** | **Mean** | **Standard Deviation** |
| --- | --- | --- | --- |
| **fixation count** | *eye play* | 299.75 | 32.00 |
|  | *observation1* | 295.31 | 62.91 |
|  | *hand play* | 285.06 | 43.10 |
|  | *observation2* | 291.00 | 67.49 |
| **fixation duration (ms)** | *eye play* | 297.42 | 36.28 |
|  | *observation1* | 303.10 | 67.74 |
|  | *hand play* | 335.56 | 78.50 |
|  | *observation2* | 330.36 | 77.60 |
| **saccade length (degrees)** | *eye play* | 2.96 | .86 |
|  | *observation1* | 2.88 | .87 |
|  | *hand play* | 2.94 | 1.29 |
|  | *observation2* | 3.02 | 1.16 |
| **total eye displacement (degrees)** | *eye play* | 887.19 | 406.69 |
|  | *observation1* | 941.12 | 512.91 |
|  | *hand play* | 824.84 | 451.36 |
|  | *observation2* | 876.52 | 483.57 |
| **% of pursuit strategy** | *eye play* | 78.54 | 23.06 |
|  | *observation1* | 78.54 | 21.03 |
|  | *hand play* | 83.75 | 27.25 |
|  | *observation2* | 77.40 | 23.89 |
