# Supplementary material for: Enhancing Sensorimotor Activity by Controlling Virtual Objects with Gaze
Source: PLoS One. 2015 Mar 23;10(3):e0121562. doi: 10.1371/journal.pone.0121562 (PMC4370397; doi:10.1371/journal.pone.0121562)
Supplement: S2 Table — Results of the paired samples t-tests for the different eye parameters. For each eye parameter, three comparisons were performed. P-values are not corrected for multiple comparisons. (DOC) [file pone.0121562.s004.doc]

| **Eye parameter** | **Pair** | **t(15)** | **p** | **d** |
| --- | --- | --- | --- | --- |
| **fixation count** | *eye play-observation1* | .267 | .793 | .09 |
|  | *hand play-observation2* | -.431 | .673 | -.11 |
|  | *eye play-hand play* | 1.487 | .158 | .40 |
| **fixation duration** | *eye play-observation1* | -.322 | .752 | -.11 |
|  | *hand play-observation2* | .220 | .829 | .07 |
|  | *eye play-hand play* | -1.998 | .064 | -.64 |
| **saccade length** | *eye play-observation1* | .333 | .744 | .10 |
|  | *hand play-observation2* | -.458 | .654 | -.07 |
|  | *eye play-hand play* | .049 | .962 | .02 |
| **eye displacement** | *eye play-observation1* | -0.989 | .338 | -.12 |
|  | *hand play-observation2* | -1.366 | .191 | -.11 |
|  | *eye play-hand play* | 1.153 | .266 | .15 |
| **% of pursuit strategy** | *eye play-observation1* | .000 | 1.000 | .00 |
|  | *hand play-observation2* | 2.402 | .030 | .26 |
|  | *eye play-hand play* | -.774 | .451 | -.21 |

.
